# Antioxidants: powering the fight against fetal hypoxia

**DOI:** 10.1098/rstb.2024.0183

**Published:** 2025-08-21

**Authors:** Kimberley J. Botting-Lawford, Katie L. Skeffington, Michael P. Murphy, Anna L. David, Dino A. Giussani

**Affiliations:** ^1^ EGA Institute for Women’s Health, University College London, London, UK; ^2^ Translational Health Sciences, University of Bristol, Bristol, UK; ^3^ MRC-Mitochondrial Biology Unit, University of Cambridge, Cambridge, UK; ^4^ Department of Physiology, Development and Neuroscience (PDN), University of Cambridge, Cambridge, UK

**Keywords:** hypoxia, high altitude, fetal growth restriction, antioxidant, MitoQ, clinical translation, pregnancy

## Abstract

Hypoxia is a common challenge in fetal development. Short-term acute episodes occur during labour owing to uterine contractions or umbilical cord compression. In response, the fetus prioritizes oxygen and nutrient delivery to the brain, heart and adrenal glands at the expense of other organs, a mechanism known as the fetal brain-sparing response. However, prolonged fetal hypoxia can occur in many conditions, including placental insufficiency, pre-eclampsia, high-altitude pregnancy and fetal congenital heart disease. Chronic hypoxia increases placental and fetal oxidative stress, triggering increased long-term cardiovascular risks in adult offspring, including hypertension and coronary artery disease. Antioxidants could potentially prevent this. However, as reactive oxygen species play a crucial role in the fetal brain-sparing response, excessive antioxidant use could weaken fetal defences against acute hypoxia, increasing the risk of hypoxic–ischaemic encephalopathy. Thus, for clinical use, an antioxidant should protect against programmed cardiovascular disease while preserving fetal brain sparing. This review summarizes preclinical evidence on the efficacy of antioxidants in preventing cardiovascular disease in the offspring of hypoxic pregnancy. We compare their effects on fetal brain sparing, highlighting the ability of the mitochondria-targeted antioxidant MitoQ to protect against programmed cardiovascular disease while preserving fetal brain sparing and outlining steps for clinical translation.

This article is part of the discussion meeting issue ‘Pregnancy at high altitude: the challenge of hypoxia’.

## 1. Introduction

During embryonic and fetal development, oxygen plays a crucial role in cellular growth, differentiation and organogenesis. Inadequate oxygen supply to the fetus can lead to compensatory mechanisms, such as increased erythropoiesis, angiogenesis and changes in autonomic nervous system function, all of which can have long-term consequences on cardiovascular health [1–3]. Hypoxia also affects the development of key cardiovascular structures, including the heart and vasculature. Studies have demonstrated that chronic hypoxia can lead to left ventricular hypertrophy, reduced cardiomyocyte endowment and endothelial dysfunction [4–9]. Additionally, epigenetic modifications, such as DNA methylation and histone acetylation, play a significant role in fetal programming [10–12]. These modifications can lead to persistent changes in gene expression related to cardiovascular function, further increasing disease susceptibility [13].

## 2. Role of oxidative stress in hypoxia-induced cardiovascular disease

A major contributor to the detrimental effects of hypoxia *in utero* is oxidative stress, which arises when there is an imbalance between reactive oxygen species (ROS) production and antioxidant defences. During hypoxia, ROS are generated by multiple processes, a major source being the mitochondria, where impaired oxygen availability leads to electron leakage and superoxide formation. Additional sources include upregulated NADPH oxidases (e.g. NOX2 and NOX4), uncoupled nitric oxide synthase, xanthine oxidase activity and endoplasmic reticulum stress (reviewed in [14]). Under normal physiological conditions, ROS play an important role in cellular signalling and homeostasis, for example, contributing to processes involved in immunity, cell differentiation and autophagy (reviewed in [15,16]). Under hypoxic conditions, an increase in ROS is also involved in mounting an appropriate physiological response—for example, via regulation of hypoxic inducible factor 1α (HIF-1α), which is an important sensor of cellular oxygen levels. In normoxia, prolyl-hydrolase enzymes target HIF-1α for degradation, however, in hypoxia, an increase in superoxide radicals inhibits prolyl hydrolase, allowing HIF-1α to activate appropriate metabolic changes such as erythropoiesis [17,18].

Under chronically hypoxic conditions, however, ROS production can become excessive, leading to detrimental effects, including oxidative damage of lipids, proteins and DNA. Oxidative stress also disrupts nitric oxide (NO) signalling, a crucial regulator of vascular tone. Reduced NO bioavailability leads to impaired vasodilatation, contributing to endothelial dysfunction and vascular remodelling, programming hypertension and atherosclerosis later in life [13,19,20]. Additionally, oxidative stress can activate inflammation, increasing the expression of pro-inflammatory cytokines and adhesion molecules, which further exacerbates endothelial damage, cardiovascular remodelling and dysfunction [21]. Reducing excessive oxidative stress through the use of antioxidants is being investigated as a potential therapy during pregnancies compromised by chronic fetal hypoxia to improve maternal and neonatal/offspring outcomes. However, ROS also play important physiological roles during normal pregnancy, crucially in the fetal defence against acute episodes of hypoxia through the fetal brain-sparing response.

## 3. The fetal cardiovascular response to acute hypoxia: the fetal brain-sparing response

The physiology mediating the fetal cardiovascular response to acute hypoxia is well characterized and involves activation of the autonomic nervous system, the release of endocrine vasoconstrictors and local redox responses at the level of the vasculature [22]. Activation of the autonomic nervous system is triggered by the carotid chemoreflex, which induces bradycardia (slow heart rate) through the vagus nerve and activates the sympathetic chain to modulate perfusion. Arterioles regulate blood flow by either decreasing (vasoconstriction) or dilating (vasodilatation) their luminal diameter, depending on physiological demand. During fetal hypoxia, activation of the sympathetic nervous system leads to peripheral vasoconstriction, which can be measured by a fall in femoral arterial blood flow [23]. By contrast, hypoxia affects cerebral arterioles directly, relaxing their smooth muscle, decreasing vascular resistance and increasing perfusion to meet metabolic demands [24]. Dilatation of the cerebral vascular bed during acute hypoxia in the fetus can be measured by an increase in fetal carotid blood flow [23]. It is the net effect of this differential vasomotion in the fetal circulation that allows a greater proportion of fetal cardiac output to be directed to the fetal brain, what is known as the fetal brain-sparing response [22].

Once triggered by a carotid chemoreflex, the peripheral vasoconstriction is maintained by the release of constrictor hormones into the fetal circulation, including catecholamines, vasopressin, neuropeptide Y and angiotensin II [22]. Furthermore, local vascular redox responses, shaped by the interaction between NO and ROS, such as the superoxide anion (O₂^•^⁻), serve as a key regulator of peripheral vasoconstriction. While NO promotes vasodilation through the relaxation of the smooth muscle in the wall of arteries, O₂^•^⁻ limits NO bioavailability by sequestering it to form the peroxynitrite molecule (ONOO⁻), which then degrades to products that react with endogenous thiol systems [25]. During acute hypoxia, both NO and O₂^•^⁻ formation are increased, but hypoxia favours an increased O₂^•^⁻ : NO ratio, thereby contributing to the fetal peripheral vasoconstriction [22]. Increased NO synthesis in the fetal circulation during hypoxia has been confirmed through using L-NAME, an inhibitor of NO synthase, which enhances the fetal femoral vasoconstrictor response to acute hypoxia [26]. Increased O₂^•^⁻ synthesis in the fetal circulation during hypoxia has been confirmed through the use of antioxidants [22,27]. Fetal treatment with antioxidants during acute hypoxia quenches the increased O₂^•^⁻ production, enhancing NO bioavailability, which weakens the fetal femoral vasoconstrictor response to acute hypoxia by opposing the chemoreflex and endocrine vasoconstrictor drive [22,27]. Thus, ROS are not simply by-products of hypoxia but play an active physiological role in regulating blood flow during hypoxic stress. Consequently, there are concerns surrounding the use of antioxidants to treat fetal hypoxia to protect against the programming of cardiovascular disease, as they may reduce O₂^•^⁻ in the fetal vasculature and, consequently, diminish peripheral vasoconstriction and thereby the fetal brain-sparing response.

## 4. Antioxidants and the modulation of the fetal brain-sparing response

**Figure 1.**
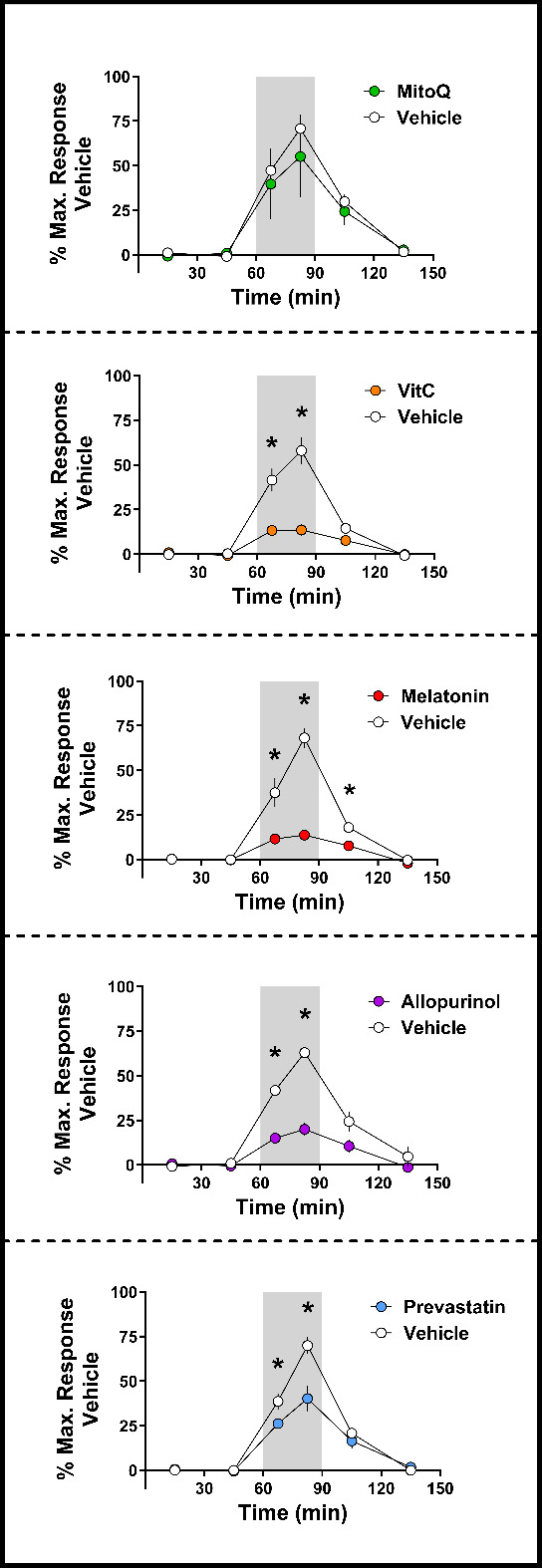
Femoral vasoconstrictor response to acute hypoxia in late gestation fetal sheep. Acute hypoxia in the late gestation sheep fetus results in an increase in femoral vascular resistance. Treating with MitoQ does not alter the hypoxia-induced femoral vasoconstrictor response; however, treatment with vitamin C (VitC), melatonin, allopurinol or pravastatin reduces it, weakening the fetal brain-sparing response to acute hypoxia. All fetuses were exposed to a 30 min acute hypoxia challenge (grey box) with either vehicle (open circles; appropriate for each antioxidant) or antioxidant exposure (coloured circles) the subsequent day. Green, MitoQ (MS010, 6 mg kg^−1^, *n* = 6; vehicle, 0.9% saline, *n* = 6; maternal IV); orange, vitamin C (0.94 ± 0.04 g kg^−1^, *n* = 8; vehicle, heparinized saline, *n* = 8, fetal IV); red, melatonin ([59] ± 9 mg kg^−1^, *n* = 6; vehicle, 0.5% ethanol in heparinized saline, *n* = 6, fetal IV); purple, allopurinol (150 mg kg^−1^, *n* = 6; vehicle, 4 M NaOH to achieve the same pH of the allopurinol solution, *n* = 6, maternal IV); blue, pravastatin (25 mg, *n* = 8; vehicle, 0.9% saline, *n* = 8, fetal IV). As this set of experiments was run over a decade, each animal’s vasoconstrictor response is reported as a percentage of its maximum femoral vasoconstriction during hypoxia when treated with a vehicle. Values are means ± s.e.m. averaged over 30 min epochs during normoxia and recovery and over 15 min epochs during acute hypoxia. Data were analysed by two-way repeated measures ANOVA for the effect of antioxidants over time. Significant (*p* < 0.05) differences are: *versus vehicle. Reproduced with permission from [27,28,31,36,53].

The importance of ROS in the regulation of peripheral vasoconstriction and the fetal brain-sparing response to acute hypoxia was first identified by the Giussani and colleagues laboratory. In these experiments, fetal sheep were exposed to acute hypoxia with and without the antioxidant vitamin C [27]. These fetuses were previously surgically instrumented with catheters and flow probes that enabled the recording of heart rate, blood pressure, blood flow and vascular resistance in the femoral artery following full post-surgical recovery. By modulating the NO to O₂^•^⁻ ratio in favour of NO, Thakor *et al*. [27] demonstrated that vitamin C blunted the hypoxia-induced peripheral vasoconstriction (table 1; figure 1) without affecting bradycardia. These results demonstrated that while O₂^•^⁻ was not required to sense hypoxia or trigger the fall in fetal heart rate, it was an essential component of the fetal peripheral vasoconstriction. Subsequently, other antioxidants and modulators of NO availability, such as melatonin [31], allopurinol [28] and pravastatin [53], have all been shown to blunt the femoral vasoconstriction when fetuses are exposed to acute hypoxia (table 1; figure 1). Importantly, blocking NO restores the magnitude of the fetal peripheral vasoconstriction in response to hypoxia in the presence of antioxidants [27,28,31,53]. This confirms that it is the balance between the dilator effects of NO and the constrictor effects of O₂^•^⁻ that acts as a vascular oxidant tone, contributing to the peripheral vasoconstriction during hypoxia and, thereby, the fetal brain-sparing response. Therefore, caution is advised when using conventional antioxidants to protect the hypoxic fetus against programmed cardiovascular disease in adulthood as a result of oxidative stress. This is because their use may weaken the fetal brain-sparing response to acute hypoxia, of the type that may occur during labour and delivery, placing the fetal brain at risk of injury and hypoxic–ischaemic encephalopathy. The ideal antioxidant would combat hypoxia-induced oxidative stress while preserving the fetal brain-sparing response. Therefore, the research effort has focused on the mitochondria-targeted antioxidant MitoQ.

**Table 1.** Effect of antioxidants on the fetal brain-sparing response to acute hypoxia and on long-term cardiovascular outcomes in offspring of hypoxic or healthy pregnancy.

antioxidant	mechanism	effect on fetal brain-sparing response to acute hypoxia	effect of antioxidant treatment on cardiac and peripheral vascular dysfunction in the fetal or adult offspring following chronic fetal hypoxia	effect of antioxidant treatment on cardiac and peripheral vascular function following healthy control pregnancy
**allopurinol**	inhibits xanthine oxidase, an enzyme responsible for producing ROS during oxidative stress. By limiting ROS production, allopurinol reduces oxidative damage	**impaired** impaired vasoconstriction of peripheral circulation in the late gestation sheep fetus [28]	**protective** improved cardiac contractility, sympathetic activity and recovery from an ischaemic challenge in the adult rat offspring [29]. Improved peripheral vasodilatation in the adolescent lamb [30]	no change in cardiac function in adult rat offspring of normoxic pregnancy [29]. Impaired dilatation of peripheral arteries compared with untreated controls at four months of age, but resolved by 15 months of age in adult rat offspring of normoxic pregnancy [30]
**melatonin**	strong antioxidant and anti-inflammatory properties. It scavenges ROS and supports mitochondrial function, which is crucial in maintaining cellular energy and reducing oxidative damage during hypoxia	**impaired** impaired vasoconstriction of peripheral circulation in the late gestation sheep fetus [31]	**protective** decreased blood pressure in the newborn lamb [32]. Improved cardiac function and endothelial- dependent vasodilatation in the late-incubation hypoxic chicken embryo [33]. Improved cardiac structure and vascular function in adult rat offspring of hypoxic pregnancy [34]	change in cardiac structure [35], but no change in peripheral vascular function in adult rat offspring of normoxic pregnancy [33,34]
**MitoQ**	a mitochondria-targeted antioxidant that specifically reduces ROS within mitochondria, protecting cells from oxidative stress and mitochondrial dysfunction during cellular hypoxia	**protected** only influences mitochondrial ROS : NO balance. Does not affect oxidant tone in peripheral arteries. Maintains the brain-sparing response in the late-gestation sheep fetus [36]	**protective** improved cardiac and vascular dysfunction programmed by hypoxic pregnancy in adult rats and in adolescent sheep [36,37]	normotensive, but enhanced dilatation of peripheral arteries to NO donor, sodium nitroprusside (SNP), in adolescent lambs born from normoxic pregnancy [36] and greater change in systolic blood pressure and peripheral blood flow to vasoconstrictor, phenylephrine in adult rat offspring of normoxic pregnancy [37]
**nMitoQ**	nanoparticle-encapsulated MitoQ that does not cross the placenta	**not known**	**protective** improved cardiac diastolic and mitochondrial function in adult female offspring of hypoxic pregnancy in rats [38,39]. Improved tolerance to cardiac ischaemia/reperfusion in adult offspring of hypoxic pregnancy in rats [40,41]. Improved vascular function in adult offspring of hypoxic pregnancy in rats [38,42]	altered cardiac diastolic function and enhanced sensitivity to vasodilator, methacholine at 13 months of age in adult rat offspring of normoxic pregnancy [38]. Males—reduced sensitivity to vasoconstrictor phenylephrine at four months, but enhanced sensitivity at 13 months of age in adult rat offspring of normoxic pregnancy [38]. Males—increased cardiac mitochondrial coupling efficiency in adult rat offspring of normoxic pregnancy [39]. No change on cardiac tolerance to ischaemia/reperfusion in adult rat offspring of normoxic pregnancy [41]
***N*-acetylcysteine (NAC**)	acts as a precursor to glutathione, one of the body’s most important endogenous antioxidants. NAC helps reduce oxidative stress by replenishing glutathione levels, scavenging free radicals and modulating redox balance	**not known**	**protective** improved endothelial function in the late incubation hypoxic chicken embryo [43] and late gestation guinea-pig fetus [44], but postnatal cardiovascular function not known	**not known**
**phosphodiesterase inhibitors**	sildenafil and tadalafil promote vascular dilatation through inhibiting phosphodiesterase−5 (PDE5), increasing Cyclic guanosine monophosphate (cGMP) and enhancing NO signalling. They can also act as an antioxidant by inhibiting NADPH oxidase activity and enhancing endogenous antioxidant enzyme activity	**impaired** effect on fetal peripheral vasoconstrictor response to acute hypoxia is not known; however, sildenafil impaired the cardiovascular adaptation to chronic hypoxia in late gestation growth-restricted fetal sheep [45]	**undefined** *sildenafil* improved vasodilatation in the late incubation hypoxic chicken embryos [46], but impaired vasodilation in growth-restricted fetal sheep [47]. Decreased fetal blood pressure and increased femoral blood flow in growth-restricted fetal sheep [45]. Postnatal cardiovascular function not known	*sildenafil* impaired vasodilation in late gestation fetal sheep from control pregnancy [47]. increased occurrence of pulmonary hypertension in human neonates from mothers who received sildenafil in the STRIDER trial [48]
**polyphenols**	resveratrol is a natural polyphenol with antioxidant properties. It also increases NO bioavailability through activation of sirtuin-1, which promotes NO production through endothelial NO synthase	**not known** maternal resveratrol treatment does not alter basal fetal haemodynamics [49]	**protective** protection against fetal demise, however, postnatal cardiovascular function is not known in a rat model of hypoxic pregnancy [50]. Improved cardiac recovery from an ischaemia/reperfusion if administered postnatally in a rat model of hypoxic pregnancy. High-fat diet [51]	**not known**
**pyrroloquinoline quinone (PQQ**)	PQQ is a redox cofactor that sequesters mitochondrial O₂^•^⁻ and other free radicals	**not known**	**protective** improved fetal cardiomyocyte endowment in spontaneously growth-restricted guinea pigs [52]	**not known**
**statins**	lowers cholesterol, but also has antioxidant properties by inhibiting NADPH oxidase, a key producer of ROS. This reduction in oxidative stress improves endothelial function and vascular reactivity	**impaired** impaired vasoconstriction of peripheral circulation in the late gestation sheep fetus [53]	**protective** enhanced NO bioavailability in the mouse term fetus [54,55]	**not known**
**vitamin C**	vitamin C is a potent antioxidant that neutralizes ROS and helps maintain endothelial function by enhancing the bioavailability of NO	**impaired** impaired vasoconstriction of peripheral circulation in the late gestation sheep fetus [27]	**protective** improved postnatal endothelial function, blood pressure and cardiac function in the adult rat offspring of hypoxic pregnancy [6,56–58]	no change in postnatal cardiac function in adult rat and sheep offspring of normoxic pregnancy [6]. However, impaired peripheral vasodilation in adult rat and sheep offspring of normoxic pregnancy [6,58]. Reduced [56] or no change in postnatal blood pressure in adult rat and sheep offspring of normoxic pregnancy [58]

## 5. Mitochondria-targeted antioxidant therapy with MitoQ

**Figure 2.**
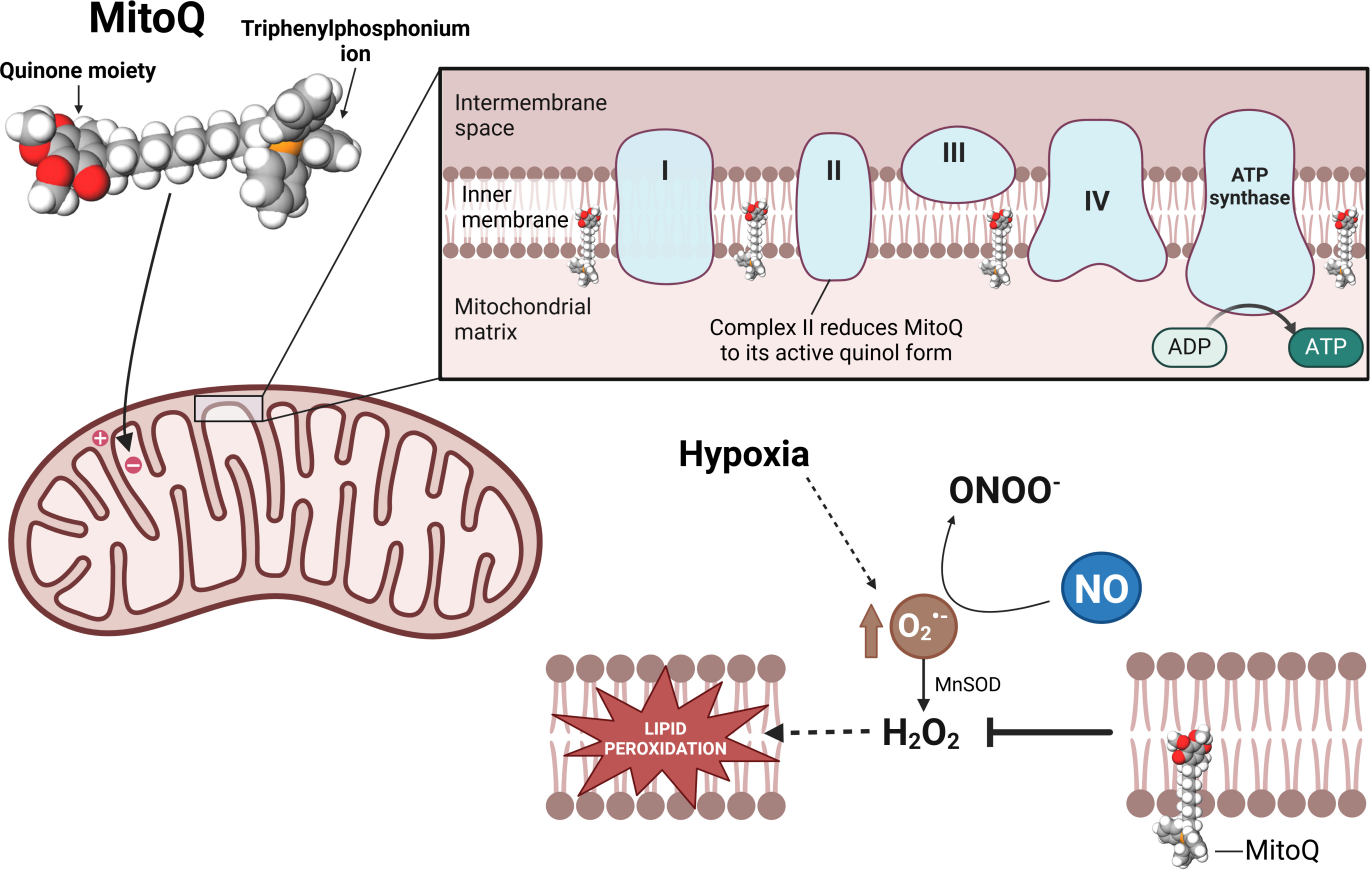
The mechanism of action of MitoQ. MitoQ (top left) accumulates in the mitochondrial matrix owing to the mitochondrial membrane potential. MitoQ embeds in the inner mitochondrial membrane, with the phosphonium ion bound at the level of the fatty acid carbonyls and the quinone moiety inserted in the hydrophobic core of the phospholipid bilayer. Under hypoxic conditions, the generation of the superoxide anion (O₂^•^⁻) by the electron transport chain increases. O₂^•^⁻ is converted into H_2_O_2_ by (manganese superoxide dismutase (MnSOD), which results in lipid peroxidation and mitochondrial damage. However, the presence of MitoQ is protective against lipid peroxidation without reacting directly with the O₂^•^⁻. This means that the reaction of O₂^•^⁻ with nitric oxide (NO) to form peroxynitrite remains unaffected. This is important because the interaction between O₂^•^⁻ and NO generates a local redox constrictor effect that contributes to the fetal peripheral vasoconstriction and, thereby, the fetal brain-sparing response to acute hypoxia. Therefore, MitoQ is the only known antioxidant that maintains the fetal brain-sparing response to acute hypoxia while preventing the developmental programming of cardiovascular disease in adult offspring of hypoxic pregnancy. Created in https://BioRender.com.

MitoQ is comprised of a triphenylphosphonium ion, covalently bound to an antioxidant ubiquinol moiety (figure 2). The cation is positively charged, but it is lipophilic owing to the dissipation of the positive charge across the surface of the phenyl groups. Therefore, MitoQ can diffuse across any lipid bilayer and is not dependent on the expression of any carrier protein. The uptake of MitoQ into mitochondria is driven by the negative mitochondrial membrane potential and leads to mitochondrial MitoQ accumulation between 100- and 1000-fold greater than achieved by non-targeted antioxidants [60,61]. Once MitoQ is accumulated within the mitochondria, the long alkyl chain sits within the hydrophobic membrane, giving the antioxidant moiety access to the lipid bilayer [60]. Within the mitochondria, MitoQ is reduced by complex II to its active ubiquinol form, which acts as a chain-breaking antioxidant, blocking H_2_O_2_-induced lipid peroxidation and apoptosis without directly regulating H_2_O_2_ production [62,63].

Hypoxia-induced lipid peroxidation results from a chain of events starting with an increase in hypoxia-induced mitochondrial O₂^•^⁻ release [64]. Some of the increased O₂^•^⁻ generated will react with circulating NO, potentiating peripheral vasoconstriction and contributing to the fetal brain-sparing response to acute hypoxia. The remaining O₂^•^⁻ will be converted to hydrogen peroxide within the mitochondria by manganese superoxide dismutase (MnSOD). The hydrogen peroxide then goes on to cause lipid peroxidation, especially in the mitochondrial inner membrane, where the unsaturated fatty acids are particularly susceptible to reacting with ROS to initiate lipid peroxidation [65]. MitoQ does not prevent the production of O₂^•^⁻ or directly interact with it to any large extent, but rather acts downstream in the chain of events by preventing lipid peroxidation and the resultant mitochondrial damage [66,67]. Therefore, O₂^•^⁻ remains available to bind NO in the peripheral circulation and maintain the fetal brain-sparing response (figure 2). Hence, MitoQ is the only antioxidant studied to date that protects against cardiovascular disease in the adult offspring programmed by fetal hypoxia without interfering with the fetal brain-sparing response to acute hypoxia [36].

## 6. Antioxidant therapy for programmed cardiovascular disease by developmental hypoxia

### (a) Vitamin C

Administration of vitamin C to the drinking water of rats undergoing hypoxic pregnancy (13% oxygen) decreased markers of fetal and placental oxidative stress and improved the cardiovascular health of the offspring in early adulthood at four months of age. Protective effects of vitamin C treatment during hypoxic pregnancy in the adult offspring included improved endothelial function, prevention of cardiac sympathetic dominance and restoration of baroreflex gain [6,56,57]. Furthermore, studies using an ovine model of improved human translational potential similarly demonstrated a protective effect of maternal intravenous treatment with vitamin C during hypoxic pregnancy on the cardiovascular health of lambs at nine months of age, including prevention of programmed systemic hypertension [58]. However, the concentration of vitamin C needed to prevent the interaction between O₂^•^⁻ and NO was unfortunately very high [68] and could promote adverse side effects, including oxaluria and kidney stones [69]. Second, clinical trials investigating the use of a lower dose of vitamin C in women at high risk of pre-eclampsia found no improvement in the incidence of pre-eclampsia, but there was a significant increase in the number of babies born with low birthweight [59,70]. Finally, vitamin C treatment also abolishes the important fetal brain-sparing response to hypoxia [27]. Therefore, although initial studies with vitamin C as a choice of antioxidant therapy provided proof of principle for the effectiveness of antioxidant treatment in protecting the adult offspring against cardiovascular disease programmed by hypoxic pregnancy, vitamin C is unfortunately not feasible for translation into clinical practice.

### (b) Melatonin

Melatonin is another antioxidant that can cross the placenta [71], and its administration by intravenous or oral routes has shown an ability to increase fetal growth, improve placental function and protect against cardiovascular dysfunction in fetal and adult offspring of pregnancy compromised by undernutrition or glucocorticoid exposure [72–77]. In the hypoxic chicken embryo, melatonin administered topically onto the chorioallantoic membrane improves cardiac contractility and endothelial function, demonstrating that it can exert protective effects through direct actions on the fetus, not just via protection against oxidative stress of the mother and/or placenta [33]. A recent study in rats investigated whether maternal oral administration of melatonin during hypoxic pregnancy (10% oxygen) was protective against cardiovascular dysfunction in the offspring in adulthood. The data showed that melatonin was protective against the development of dilated cardiomyopathy, alterations in cardiac endothelial nitric oxide synthase (eNOS) expression and vascular dysfunction in the rat adult offspring at four months of age [34]. Melatonin also improved cardiomyocyte calcium handling in hypoxic rat pregnancy (13%) [35]. Importantly, these effects of melatonin were apparent at doses less than those recommended for the short-term treatment of jetlag, highlighting that melatonin treatment could be more of a suitable candidate for clinical translation than vitamin C. However, melatonin treatment not only weakens the fetal brain-sparing response to acute hypoxia [31], but concerns have accumulated around the safety of its long-term administration. Melatonin is an endogenous molecule that affects the transcription of many genes and many physiological functions, including the regulation of circadian rhythmicity [76]. Long-term administration of melatonin to pregnant women is therefore not currently recommended owing to a lack of safety studies and potential chrono-disruptive effects on the mother and fetus [78].

### (c) Allopurinol

Some antioxidants aim to prevent the production of O₂^•^⁻ by specific pathways rather than aiming to remove ROS following their formation. Production of ROS by xanthine oxidase (XO) greatly increases under chronic hypoxia, and the antioxidant allopurinol can be used to prevent this [79,80]. Maternal oral administration of allopurinol to rats undergoing hypoxic (13% oxygen) pregnancy prevented the associated programmed increase in cardiac sympathetic dominance and impairment of cardiac function recovery from an ischaemic challenge [29]. A clinical trial investigating the effect of allopurinol administration during suspected hypoxic pregnancies on neurological outcomes of the offspring has shown no significant effects [81]. The safety of allopurinol administration during pregnancy has been debated owing to possible reports of teratogenicity, although recent studies have found little evidence to support this [82,83]. Interestingly, late gestation fetal sheep treated with allopurinol increased umbilical blood flow, suggestive of a basal XO-derived oxidant tone that limits umbilical perfusion under basal conditions [84]. However, during acute hypoxia, fetal sheep exposure to allopurinol diminished the fetal brain-sparing response [28], again reducing the impetus for this antioxidant strategy to be chosen for clinical translation.

### (d) Statins

Statins have their antioxidant effect through increasing NO bioavailability [85]. Although there were initial concerns about embryotoxicity, observational data have demonstrated an absence of adverse pregnancy outcomes with inadvertent statin use [86]. Studies in the chicken embryo have reported that pravastatin treatment via the chorioallantoic circulation during hypoxic incubation increased NO bioavailability and improved NO-dependent vasodilatation [54]. Consistent with these data, studies using oral pravastatin treatment in a rodent model of pre-eclampsia found an increase in eNOS expression in the vasculature [55]. While initial studies suggested a promising ability of statins to reduce biomarkers found to be raised in pre-eclampsia, such as sFlt-1 and chemerin [87], recent clinical trials investigating the use of oral pravastatin in pregnancies with or at high risk of pre-eclampsia showed no beneficial effects including no reduction in these biomarkers [88,89]. The lack of data supporting the beneficial effect of statins when used in pregnancy, combined with their effects to weaken the fetal brain-sparing response [53], again, is currently prohibitive against their use in clinical practice.

### (e) Phenols

Another antioxidant of interest is resveratrol, a naturally occurring phenol found in red wine that upregulates mitochondrial superoxide dismutase expression [90]. In preclinical studies using ovine pregnancy, subcutaneous administration of resveratrol increased uterine blood flow and improved fetal growth during healthy pregnancy [91] without effect on fetal haemodynamics when given as a bolus directly into the fetal femoral vein [49]. Additionally, resveratrol does not cross the placenta [49] and has been tested in several different animal models of complicated pregnancy (reviewed in [92]). Findings include dietary resveratrol improving fetal survival in a rodent model of chronic prenatal hypoxia [50] and improved cardiac function and recovery from an ischaemic reperfusion challenge in adult rat offspring who had been challenged with chronic fetal hypoxia combined with a high-fat diet post-weaning [51]. However, the safety of resveratrol treatment during pregnancy was questioned in a non-human primate study, which found concerning changes in fetal pancreatic development following maternal oral resveratrol administration [93]. Similarly, the results of clinical trials testing resveratrol in a variety of conditions have been contradictory (reviewed in [94]), with some studies finding benefits but others reporting adverse effects. The effects of resveratrol on the fetal brain-sparing effect remain unknown. Therefore, more research is needed to evaluate the efficacy of resveratrol treatment in pregnancy.

### (f) *N*-acetylcysteine

It has been suggested that exogenous antioxidant treatment or agents that increase NO bioavailability may have too slow a reaction rate to protect against pro-oxidant mechanisms [95]. Therefore, an alternative approach may be to enhance the synthesis of endogenous antioxidant mechanisms at their very site of physiological production to protect against cardiovascular disease programmed by developmental hypoxia. One molecule of interest in this regard is *N*-acetylcysteine (NAC), which has been trialled clinically in human pregnancies with impending preterm birth affected by intrauterine infection and/or inflammation, showing improved neonatal outcome after short-term intravenous administration [96,97]. However, evidence on antenatal use in pregnancies at risk of chronic hypoxia is currently lacking, and a trial of NAC in women with severe pre-eclampsia showed no benefit [98]. The effect of NAC on the fetal brain-sparing response remains unknown. However, NAC reduced the pulsatility index in the umbilical artery of fetal guinea pigs exposed to chronic hypoxia induced by progressive uterine artery occlusion [44]. The precise molecular mechanisms of the antioxidant properties of NAC are uncertain. Nevertheless, it is thought to work partly as a precursor to the production of the antioxidant glutathione, as well as promoting free radical scavenging through its conversion to hydrogen sulfide and sulfane sulfur species [99–101]. Hydrogen sulfide is also an endothelial-derived vasorelaxant with similar effects to NO [102]. A recent study has shown that both human fetuses and chronically hypoxic chicken embryos with growth restriction have increased expression of the gene *CTH*, which is involved in hydrogen sulfide synthesis [43]. Furthermore, treatment with NAC restored impaired endothelial function in both human chorionic arterial segments from growth-restricted pregnancies and the femoral artery of the chronically hypoxic chicken embryo [43].

### (g) Phosphodiesterase inhibitors

The phosphodiesterase-5 inhibitor sildenafil has been investigated as a therapy for pregnancies complicated by placental insufficiency and fetal growth restriction. Sildenafil inhibits O₂^•^⁻ production [103], NADPH oxidase ROS production [104] and enhances NO signalling [105]. Administration of sildenafil to the hypoxic chicken embryo via the chorioallantoic circulation improved peripheral vascular function, reduced measures of oxidative stress and improved NO bioavailability [46]. However, the effect of sildenafil administration during chronic hypoxia on the programming of adult cardiovascular disease has not been studied. Similarly, the effects of sildenafil administration on the fetal brain-sparing response during an acute hypoxic challenge have not been investigated, although an ovine study in late gestation demonstrated that sildenafil infusion increased fetal femoral blood flow during chronic hypoxia, suggesting possible impairment of fetal brain sparing during chronic hypoxia [45].

Early studies suggested that sildenafil might protect fetal growth in human [106], ovine and rodent [107–109] pregnancies affected by fetal growth restriction. However, a series of international randomized trials (Sildenafil TheRapy In Dismal prognosis Early onset fetal growth Restriction [STRIDER]) disappointingly found no delay in gestational age at birth or improved birthweight in treated pregnancies affected by early-onset fetal growth restriction and abnormal umbilical artery Doppler blood flow velocity. Of concern, there was an increased risk of pulmonary hypertension in neonates of mothers who received sildenafil treatment (18.8% versus 5.1% in the placebo group, *p* = 0.008), and the Dutch STRIDER trial was terminated early for this reason [48,110–113]. More recently, clinical trials in Japan are studying the longer acting phosphodiesterase-5 inhibitor tadalafil [114,115], which has a half-life of 14−15 versus 2−4 h for sildenafil. The initial phase IIa TheTADAlafil treatment for Fetuses with early-onset growth Restriction (TADAFER) placebo double-blind randomized placebo control trial recruited women with fetal growth restriction up to 34 weeks of gestation, but only 5% had abnormal umbilical artery Doppler blood flow velocity, and 25% had abnormal uterine artery Doppler blood flow velocity, a very different population to those recruited to the STRIDER trials [116]. Tadalafil decreased the perinatal death rate, possibly via prolonging pregnancy, and tadalafil decreased the umbilical artery pulsatility index when the estimated fetal weight was very small (−2 s.d.). The results of the larger TADAFER IIb study await completion and analysis (Cochrane Central Register of Controlled Trials CN-01970876).

### (h) MitoQ

Mitochondria are an attractive target for antioxidant treatment of complicated pregnancy because mitochondria are a major source of cellular oxidative stress [117], and the production of mitochondrial ROS is known to increase under hypoxic conditions [64]. Treating mitochondrial O₂^•^⁻ with oral pyrroloquinoline quinone improved fetal cardiomyocyte endowment in spontaneously growth-restricted guinea pigs [52]; however, its effect on the fetal brain-sparing response is unknown. Other conventional antioxidant therapies are largely ineffective against mitochondrial oxidative stress as only a small proportion is able to penetrate mitochondria. However, MitoQ, which readily accumulates in the mitochondria, is an antioxidant which specifically targets mitochondria-derived oxidative stress without preventing the O₂^•^⁻ and NO reaction required for the brain-sparing response [36,61]. Preclinical studies found that maternal oral and intravenous administration of MitoQ can cross the placenta and reach a therapeutic concentration in fetal tissues [36]. MitoQ administered to the hypoxic chicken embryo via the chorioallantoic circulation improved vascular and cardiac function, while intravenous MitoQ administration to hypoxic pregnant sheep prevented the programming of hypertension in the adult offspring via enhanced NO signalling pathways [36]. Studies in rats have also shown improved cardiovascular function in adult offspring of hypoxic pregnancies treated with oral MitoQ, including protection against increased α_1_-adrenergic reactivity, cardiac sympathetic dominance and improved peripheral vasodilatation [37,38]. Of note, encapsulating MitoQ in nanoparticles (nMitoQ) that enable MitoQ to specifically treat oxidative stress within the placenta, but not the fetus, appears to also protect the fetus exposed to maternal hypoxia from future cardiovascular disease [38–42]. MitoQ has passed through formal animal toxicity, human phase 1 studies, and three published phase 2 studies in non-pregnant adults with no toxicity reported [118]. Additionally, MitoQ has also been available as an oral supplement since 2013, with no toxicity reported at the oral doses recommended [119]. The combination of MitoQ being the only antioxidant studied to date that does not impair the fetal brain-sparing response to hypoxia [36], coupled with it being a drug already approved for use in human clinical trials to test its safety and efficacy under various disease states [61], makes it an attractive candidate for human clinical translation for protecting cardiovascular dysfunction in adult offspring programmed by hypoxic pregnancy.

## 7. The path to clinical translation

There are challenges when taking maternal antioxidant therapy into clinical practice to target fetal origins of an increased cardiovascular risk in offspring. This is most difficult when considering therapeutics that may be untested in pregnancy or even novel to human use. First, the mantra for prenatal therapy is safety first [120]. The trial team must consider not only the safety of the mother and her current fetus as well as the future neonate, child and adult the fetus would become but also the effects on any future sibling offspring. For instance, via an impact of the therapy on the maternal ovaries and thereby her future reproductive capacity, or transgenerational consequences through effects on the fetal primordial follicles if the fetus is female. Safety is commonly assessed in preclinical animal models through toxicology and/or reproductive toxicology studies, with specific guidelines available on the types of studies, including high dose exposure, length of time and expected analyses [121]. The development of *in silico* computational models to predict *in vivo* drug effects and *ex vivo* placental perfusion to assess short-term drug safety goes some way to reducing animal use according to 3Rs principles [122], but cannot completely replace them in the current International Council for Harmonization guidance.

Second, the therapy must be effective by improving fetal/neonatal outcome, and if possible, by improving maternal health or certainly without detriment to her well-being. Preclinical models that can optimally mimic developmental programming by chronic fetal hypoxia in humans are required, but no one model species is able to fully recapitulate this. Genetically modified mice offer a well-characterized phenotype/genotype, and rats are useful from a regulatory perspective and have a greater size for manipulation. However, guinea pigs have the most similar rodent placenta to humans [123,124]. Sheep are very well understood for fetal physiology and amenable to surgical instrumentation for long-term physiological recording [125]. Although expensive and with ethical concerns, non-human primates are commonly used for safety testing, providing data for regulatory submissions [126].

Another concern is that the harm in the adult offspring associated with fetal programming from chronic hypoxia can take many years and sometimes decades to manifest, rendering trials unfeasible and prohibitively expensive. Therefore, short-term surrogate markers of safety and effectiveness are needed that are closely associated with the fetal programming effect, but which can be detected optimally either *in utero* or at/soon after birth. Another reason to have a short-term surrogate marker is that in a first-in-human clinical trial of a novel therapeutic, patient dosing and dose escalation may be advised serially, with safety/effectiveness observed after each patient, before moving to the next patient/higher dose. Short-term fetal endpoints can be difficult to assess. Direct quantification of fetal oxygen saturation or fetal cardiac remodelling is currently not possible, but changes in fetal growth velocity, cardiac function and circulatory redistribution can be observed using ultrasound and Doppler blood flow velocimetry [127]. Therefore, triallists may need to rely on neonatal circulating protein markers of neural and cardiac injury, such as S100b [128] and troponin T [129], respectively.

Testing existing drugs used for other purposes, namely drug repurposing, offers a potentially shorter route to market. Identification of the drug candidate increasingly uses computational and experimental approaches such as artificial intelligence algorithms and other bioinformatics tools. Repurposed drugs have the advantage of already having passed many safety and toxicology tests, and there may be data on drug use in pregnancy via post-marketing surveillance.

**Figure 3.**
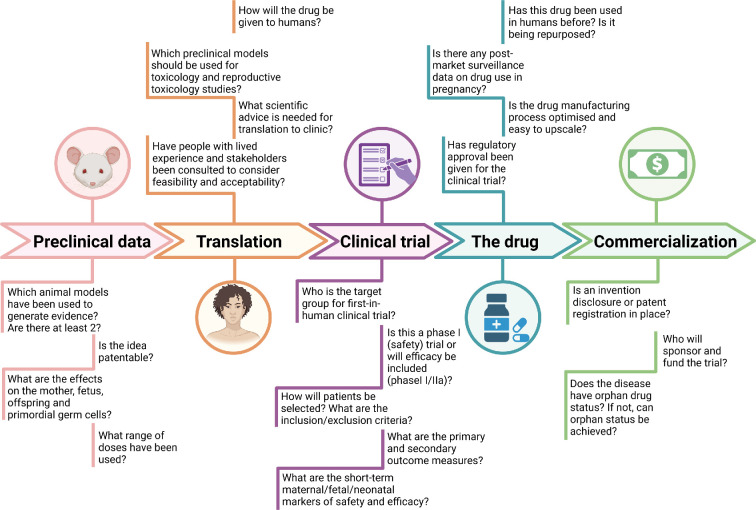
Path to clinical translation. A schematic representation of the key stages in drug development, from preclinical research to commercialization. The process is divided into five phases: preclinical data, translation, clinical trial, the drug and commercialization. Each phase is associated with critical questions specific to treating fetal hypoxia that guide decision-making. In the preclinical stage, essential considerations include the selection of animal models, dose range and potential effects on maternal and fetal health. The translation phase addresses the feasibility of clinical application, regulatory guidance and stakeholder engagement. The clinical trial phase focuses on study design, target population, safety and efficacy markers. The drug phase involves regulatory approval, manufacturing optimization and prior human use. Finally, the commercialization phase ensures patent protection, funding and market positioning, including orphan drug status. This framework highlights the multidisciplinary approach required for successful drug development. Created in https://BioRender.com.

Once initial efficacy is demonstrated, the potential route to market follows a well-trodden path and includes scientific advice with regard to toxicology/reproductive toxicology study plans and drug manufacture/quality testing (figure 3). Clinical trial design must include identifying the population at risk to define the inclusion and exclusion criteria for the protocol. Target product profiles (TPPs, such as those recently developed for the prevention and treatment of pre-eclampsia, are important tools to drive new drug development by specifying upfront the characteristics that new products should take [130]. To date, no TPP exists for fetal hypoxia. For early phase trials where drugs are being used for the first time in humans, dose-escalation studies are usually performed. The baseline adverse event rate of the maternal and fetal condition should be well understood ahead of drug testing to assist with determining the acceptable toxicity level and to balance the risk versus benefit for the drug in the at-risk population [131]. Fetal hypoxia may occur via a number of mechanisms such as placental insufficiency or fetal structural anomalies; therefore, choosing the population in whom to test the drug requires careful thought. Engagement with the patient population and stakeholders and involving them early during the clinical translation pathway is strongly recommended. Patients can become advocates of the need for therapy; they provide information on what level of harm may be acceptable to them and improve decision-making about the ethics of a drug treatment, clinical trial participation and trial outcome measures [132]. Their view may also be pivotal in getting clinical trial ethical approval. Phase III trials often include an element of randomizing patients to a treatment or placebo group to reduce the risk of bias. Getting patient input into decisions as to whether randomization is possible and how it might be achieved (e.g. low versus high dose drug, early versus delayed randomization) is critical to ensure that a trial recruits well.

Invention disclosure and patent registration are useful to commercialize a therapeutic approach, but the length of time to develop a drug, particularly with relevance to maternal and fetal indications, may mean that there is limited time left on the patent by the time it reaches the market. Securing orphan drug designation will smooth the regulatory submissions, and in Europe, for example, it provides 10 years of market exclusivity to protect companies from competition, which can aid the recovery of expensive research costs [133]. A recent estimate for developing a new drug found that the cost in the last 20 years was approximately $173 million (range, $73 million for genitourinary to $298 million for pain and anaesthesia), inclusive of post-marketing studies, increasing to $516 million when the cost of failures was included [134]. The cost of drug development for a maternal and fetal condition is likely to be higher given the complexity and extra challenges, although the need is greater than for many paediatric or adult illnesses.

## 8. Summary

In conclusion, the interaction between O₂^•^⁻ and NO at the level of the vasculature plays a critical role in the regulation of fetal peripheral vasoconstriction, which contributes to the fetal brain-sparing response to acute hypoxia. While conventional antioxidants weaken the fetal peripheral vasoconstriction, the mitochondria-targeted antioxidant MitoQ has emerged as a promising candidate for protecting against the long-term adverse cardiovascular consequences of fetal hypoxia without impairing the fetal brain-sparing response to acute hypoxia. However, translating maternal antioxidant therapy into clinical practice presents several challenges, including the identification of reliable short-term surrogate markers to facilitate early-stage clinical trials. Repurposing existing drugs, such as MitoQ, offers a potential pathway to accelerate clinical translation, as these compounds have already undergone extensive safety evaluations, including human clinical trials. However, further research is required to establish definitive safety and efficacy profiles specific to its treatment during pregnancy. Future research efforts should focus on refining antioxidant therapies, identifying reliable biomarkers for fetal programming and establishing robust clinical trial frameworks. Addressing these challenges will be essential in developing effective strategies to improve long-term cardiovascular health outcomes for individuals exposed to fetal hypoxia.

## Data Availability

All data presented in this review have been previously published and can be sourced from the following articles: MitoQ; [36], vitamin C; [27], melatonin; [31], allopurinol; [28], pravastatin; [53].
